# Maternal mediation strategies during interaction with toddlers- a comparison of dyads with autism spectrum disorder (ASD) and dyads with typical development (TD)

**DOI:** 10.1192/j.eurpsy.2021.1687

**Published:** 2021-08-13

**Authors:** A. Mimouni-Bloch, A. Oren, E. Dromi

**Affiliations:** 1 Sackler Faculty Of Medicine, Tel Aviv University, Tel Aviv, Israel; 2 Child Development Center, Loewenstein Rehabilitation Center, Raanana, Israel; 3 Constantiner School Of Education, Tel Aviv University, Tel Aviv, Israel

**Keywords:** toddlers, autism, Dyadic naturalistic interaction, early language

## Abstract

**Introduction:**

During interactions with toddlers, mothers use various mediation strategies to encourage mutual play. Such mediation skills play an important role in the development of toddlers’ communicative skills. Autism Spectrum Disorder (ASD) introduces challenges to this interaction.

**Objectives:**

To study the use of maternal strategies during interaction with ASD and TD toddlers at early lexical levels.

**Methods:**

Nine ASD and fifteen TD dyads participated. Toddlers were matched by lexical levels. The mean age in the ASD was 31.5 months and in TD - 17 months. Each dyad was video-recorded three times, during naturalistic interaction. Mothers’ verbal mediation strategies were divided into five main communicative categories.

**Results:**

1. Exact repetition of toddler’s utterances was similarly used and increased in both groups across the three visits (f (2,44)=3.77, p< 0.05). 2. Significant differences were found between the two groups regarding strategies associated with control of the interaction eg mothers of toddlers with ASD (MASD) made more frequent attempts to redirect their child’s attention (F (1,22)= 74.56, p<0.01). 3. MASD had higher indices of overall talkativeness (F (1,22)= 5.43, p<0.05); use of nonverbal means (F(1,22)= 9,51, p<0.01); simultaneous use of different means of communication (F (1,22)=19.8, p<0.01).

**Conclusions:**

Our results highlight that in some respects, maternal mediation strategies reflect the child’s lexical level. However, our main finding is a distinct interaction style expressed in MASD’s elevated use of verbal and nonverbal mediation strategies. This, in hope of maintaining continuous interaction that could not be otherwise achieved due to their toddlers’ difficulties

**Disclosure:**

No significant relationships.

